# HRST: An Improved HRNet for Detecting Joint Points of Pigs

**DOI:** 10.3390/s22197215

**Published:** 2022-09-23

**Authors:** Xiaopin Wang, Wei Wang, Jisheng Lu, Haiyan Wang

**Affiliations:** 1 Key Laboratory of Smart Farming for Agricultural Animals, Ministry of Agriculture and Rural Affairs, College of Informatics, Huazhong Agricultural University, Wuhan 430070, China; 2 Shenzhen Institute of Nutrition and Health, Huazhong Agricultural University, Wuhan 430070, China

**Keywords:** deep learning, object detection, keypoint detection, transformer, CNN

## Abstract

The body size of pigs is a vital evaluation indicator for growth monitoring and selective breeding. The detection of joint points is critical for accurately estimating pig body size. However, most joint point detection methods focus on improving detection accuracy while neglecting detection speed and model parameters. In this study, we propose an HRNet with Swin Transformer block (HRST) based on HRNet for detecting the joint points of pigs. It can improve model accuracy while significantly reducing model parameters by replacing the fourth stage of parameter redundancy in HRNet with a Swin Transformer block. Moreover, we implemented joint point detection for multiple pigs following two steps: first, CenterNet was used to detect pig posture (lying or standing); then, HRST was used for joint point detection for standing pigs. The results indicated that CenterNet achieved an average precision (AP) of 86.5%, and HRST achieved an AP of 77.4% and a real-time detection speed of 40 images per second. Compared with HRNet, the AP of HRST improved by 6.8%, while the number of model parameters and the calculated amount reduced by 72.8% and 41.7%, respectively. The study provides technical support for the accurate and rapid detection of pig joint points, which can be used for contact-free body size estimation of pigs.

## 1. Introduction

Livestock body size is an important indicator for growth status assessment, body weight estimation, and selective breeding of pigs [1,2,3]. Therefore, the accurate measurement of pig body size is of great significance to the scientific management of farms. Traditional measurement methods require close contact between humans and animals, which is inefficient, inaccurate, and often causes stress to pigs. With the advance of deep learning and machine vision, non-contact, fast, accurate, and non-stressful measurement methods are emerging in the livestock industry. Pezzuolo et al. [4] used an image-processing algorithm to extract measurement points to estimate the body size of pigs. Zhang et al. [5] extracted feature points based on the concavity and convexity of the contour to estimate the sheep body size. Wang et al. [6] used the target contour extracted by the U-net segmentation model combined with the binocular ranging to estimate the yak body size. However, these methods are complicated because their successful implementation depends on two steps: first, the extraction of the target contour, and then, the location of measurement points. In addition, they are only suitable for a single target in a specific scene, and the measurement accuracy is easily affected by illumination, which significantly limits its application to large farms. By contrast, multi-target keypoint detection methods based on deep learning can directly locate the joint points and has stronger robustness and generalization ability, providing new insights to the detection of measurement points.

Multi-target keypoint detection methods can be divided into top-down and bottom-up. The top-down approach first detects a series of object instances with bounding boxes and then performs keypoint detection on each object instance. On the contrary, the bottom-up method directly identifies the key points of all object instances in the image and then groups them into different objects [7]. Compared with the bottom-up method, the top-down method can normalize the objects to the same size according to the detected object bounding box, so it is generally insensitive to the scale variation and has higher detection accuracy [8]. Currently, the implementation of top-down keypoint detection methods relies on object detection and keypoint detection methods.

Object detection is the first step in top-down keypoint detection methods. It can be divided into one-stage and two-stage according to whether to generate region proposals. The one-stage target detection algorithm directly generated object bounding boxes with class labels. By contrast, the two-stage model first generated a series of region proposals containing the target and then classified and regressed each region proposal to obtain a more accurate bounding box [9]. The one-stage detection model can be divided into the anchor-free and anchor-based detectors based on whether the anchor needs to be preset. YOLOv4 [10] is an anchor-based detector, which uses CSDarknet53 as the feature extraction network, and introduces the SPP module and PAN module based on YOLOv3 [11] to increase the receptive field and learn rich semantic information. An anchor-free detector, named CenterNet [12], converts the object detection into the center point estimation and obtains the object bounding box by predicting the offset, width, and height of the object’s center point. FCOS [13] is a fully convolutional anchor-free detector, which utilizes all points in a ground truth bounding box to predict the bounding boxes and adds a center-ness branch to suppress the generation of low-quality prediction bounding boxes. In two-stage detection models, Faster-RCNN [14] is the most representative algorithm. It introduces Region Proposal Network (RPN) based on Fast R-CNN [15] and realizes end-to-end training, which can significantly improve the detection speed of the model while maintaining accuracy. In addition, the oriented detectors can more accurately represent the target position, which has also received extensive attention from researchers. For example, RoI Transformer [16] combines RRoI Learner and RRoI Warping to implement oriented object detection. Cheng et al. [17] proposed an Anchor-free Oriented Proposal Generator (AOPG) to generate high-quality oriented proposals.

As the second step of top-down keypoint detection, several keypoint detection frameworks have been proposed. For example, Chen et al. [18] proposed a Cascaded Pyramid Network (CPN) to locate difficult joint points by fusing feature representations of different resolutions with online hard keypoint mining loss. To simplify the model structure and improve the detection accuracy, Xiao et al. [19] presented a Simple Baseline by adding several transposed convolution layers behind ResNet [20]. However, this recovering high resolution from low resolution tends to lose position information and cannot obtain optimal detection accuracy. To learn feature representations with strong location sensitivity, a High-Resolution Network (HRNet) [21] was designed by maintaining high resolution throughout the entire model structure and continuous cross-feature fusion. Since HRNet only considers features at the highest resolution, HRNetv2 and HRNetv2p [22] were developed based on HRNet to learn richer representations. Nevertheless, this convolutional neural network-based method can learn visual representations well but cannot accurately capture the constrained relationship between key points. To better learn the constraint relationship between joints, Li et al. [23] proposed Tokenpose by introducing a Transformer block based on HRNet. These joint point detection methods take a big step towards achieving state-of-the-art detection accuracy. However, two problems remain to be addressed: detection speed needs to be increased and model parameters have to be reduced.

Therefore, in this study, we proposed HRNet with Swin Transformer block (HRST) based on HRNet, aiming to increase detection speed, reduce the model parameters, but maintain high detection accuracy. HRST inherits the multi-scale fusion and high-resolution representation of HRNet, and the shifted window-based self-attention mechanism of the Swin Transformer, which makes it possible to reduce model parameters at the same time as possessing high detection accuracy. Beyond that, we implemented joint points detection of multiple pigs based on a top-down approach. Firstly, CenterNet with DLA-34 [24] as a feature extraction network was used to detect the posture of the pig (standing or lying). Then, HRST was used to detect the joint points of the pig in a standing state. Finally, we validate our method on the pig joint points test dataset and demonstrate superior detection performance. Specifically, HRST achieves an average precision (AP) of 77.4%. Compared with HRNet, the AP improved by 6.8%, while the number of model parameters and the calculated amount reduced by 72.8% and 41.7%, respectively. It also indicates that HRST can accurately and quickly locate the joint points of pigs, which can be applied to achieve non-contact, stress-free, and rapid body size estimation of multiple pigs on large-scale farms.

## 2. Materials and Methods

This section introduces the data used in the experiment and the multi-pig joint points detection method. As we know, the back of the pig in the lying posture is easily occluded, which affects the detection of joint points. To solve this problem, the multi-pig joint point detection method follows a top-down framework. Likewise, we introduce our work following the top-down framework in Section 2. We first introduce the pig posture detection method based on the object detection algorithm and then describe our improved joint detection model HRST. Finally, we combine the posture detection algorithm with HRST to locate the joint points of multiple pigs.

### 2.1. Datasets Collection and Dataset Annotation

The video surveillance data were collected from the Breeding Swine Quality Supervision and Testing Center, Wuhan, China from July to November 2021. The video acquisition equipment was Hikvision DS-2CD3346FWDA3-1 cameras. Each video had a resolution of 2560 × 1440 and was captured at a frame rate of 25 frames per second. Three pig pens were selected for the experiment, each with about ten live pigs.

**Figure 1:**
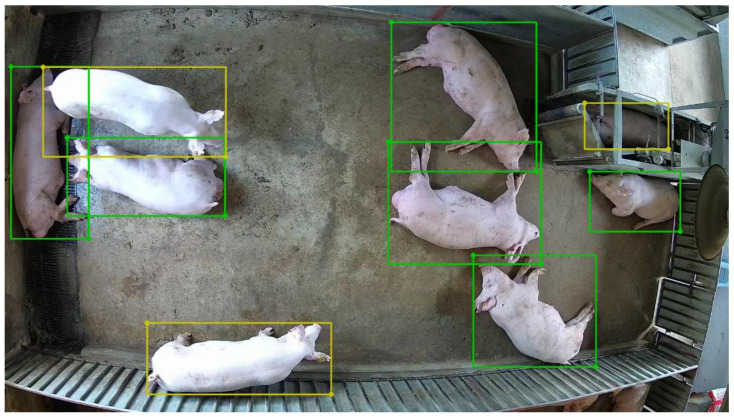
Annotated example for posture detection (the yellow bounding box represents the standing pig, and the green bounding box represents the lying pig).

Two datasets were annotated to realize multi-pig joint points detection. One was a pig posture detection dataset, and the other was a pig joint points detection dataset. For the pig posture detection dataset, 2033 images were selected by extracting a frame from surveillance videos every two seconds. Similar frames were removed. Then, we labelled the posture of those pigs as either standing or lying with the LabelMe annotate tool [25]. The labels included the information of the posture category and position of the pig. After the dataset was annotated, the training set (1627 images), validation set (203 images), and test set (203 images) were randomly sampled according to the ratio of 8:1:1. An example of pig posture labeling is shown in Figure 1, where green stands for lying, and yellow represents standing. The details of the posture detection dataset are shown in Table 1.

**Figure 2:**
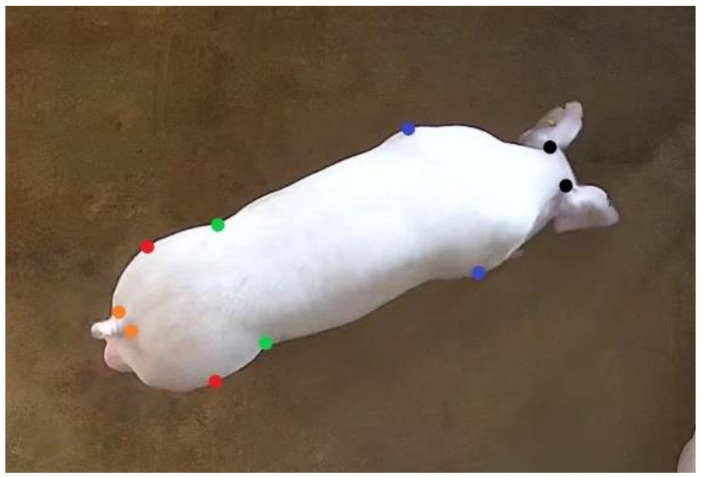
Pig joint point annotation example. (Black marks indicate left and right neck, blue marks indicate left and right shoulders, green marks indicate left and right abdomen, red marks indicate left and right hips, and orange marks indicate left and right tails).

After posture detection annotating, 1623 standing pig images were selected to locate the joint points of pigs. For the standing pigs, ten joint points of each were annotated by the LabelMe annotation tool: left neck, right neck, left shoulder, right shoulder, left abdomen, right abdomen, left hip, right hip, left tail, and right tail. As can be seen in Figure 2, the joint points are marked by different colors. After the dataset was annotated, the training set (1299 images), validation set (162 images), and test set (162 images) were randomly divided in a ratio of 8:1:1. Next, 1000 images were randomly selected from the annotated training set for second annotation to calculate the standard deviation. The standard deviation of each joint point relative to the object scale was computed for subsequent computation of Object Keypoint Similarity (OKS) [26]. The standard deviation (Table 2) of shoulders and hips is relatively large because the feature areas are not as prominent as those of the neck, abdomen, and tail, making it difficult to label.

In addition to our annotated pig joint point dataset, we used the publicly available Amur Tiger Re-identification in the Wild (ATRW) dataset [27] to further verify the generalization and transferability of the model. This dataset collects surveillance videos from multiple wildlife parks, including more than 8000 video clips from 92 Amur tigers, with bounding-box, keypoint-based pose, and identity annotations.

### 2.2. Pig Posture Detection Based on Object Detection Algorithm

Deep learning has shown its great advantages in various vision tasks such as object detection [28], crowd counting and localization [29], and keypoint detection [30]. As the first step of multi-target joint point detection, a deep learning-based object detection algorithm is used to detect the posture of pigs. In this study, we used a one-stage model and a two-stage model to detect pig posture. In the one-stage model, we compared anchor-based (YOLOv4) and anchor-free (FCOS, CenterNet) detectors. In the two-stage model, we used four different feature extraction networks, ResNet50 [20], MobileNetV3-Large [31], EfficientNetV2-S [32], and ConvNeXt-T [33] to compare the impact of different feature extraction networks on the model detection accuracy. ResNet, a residual neural network proposed by He et al. [20], can effectively alleviate the model degradation problem by introducing a residual block. MobileNetV3 [31] is a lightweight model designed for mobile devices. It inherited the depthwise separable convolution (DSC) of MobileNetV1 [34] and the inverted residual structure of MobileNetV2 [35] and introduced the squeeze-and-excitation (SE) block and h-swish activation function to improve detection accuracy. EfficientNetV2 [32] is a convolutional neural network with faster training speed and higher parameter efficiency, which introduces the Fused-MBConv module and progressive learning method based on EfficientNetV1 [36]. ConvNeXt [33] is a redesigned model of ResNet based on the vision transformer structure, which can significantly improve the model detection accuracy while maintaining the simplicity and efficiency of standard ConvNets. In addition, Feature Pyramid Network (FPN) [37] was added to the feature extraction network because it can not only generate a region proposal on multiple feature layers but also learn richer semantic information by fusing the representations of multiple feature layers.

### 2.3. HRST (HRNet with Swin Transformer Block)

**Figure 3:**
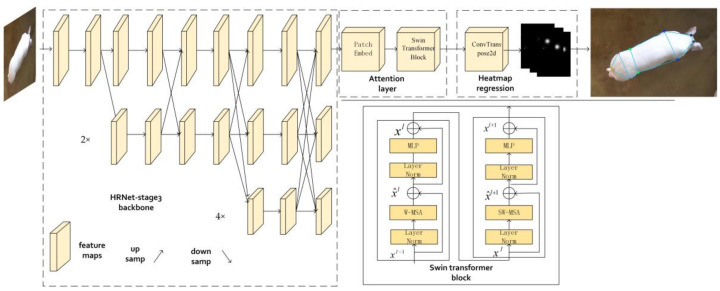
HRST model structure.

An excellent model should have high detection accuracy, fast detection speed, and relatively few model parameters. However, current joint points detection research focuses on improving the model accuracy and ignores the model parameters and detection speed. Therefore, we proposed an HRNet with Swin Transformer block (HRST) for the location of the joint points of pigs. HRST replaced the fourth stage of parameter redundancy in HRNet with a Swin Transformer block, making it possible to keep high accuracy with fewer parameters. Figure 3 shows that the HRST model is composed of three parts: HRNet-stage3, attention layer, and heatmap regression module. The details of each part are described as follows.

#### 2.3.1. HRNet-Stage3

HRNet is a network that learns strong location-sensitive high-resolution representation, which has been applied to various vision tasks such as pose estimation [38], object detection [39], and semantic segmentation [40]. It differs from previous networks that pass low-resolution features through dilated convolution or up sampling to recover high-resolution representations. Instead, it maintains high-resolution representations throughout the model process and continuously performs cross-scale fusion to learn richer representations. HRNet takes the high-resolution subnetwork as the first stage and then gradually adds a new subnetwork in parallel to form a new stage, where the resolution of the current subnetwork is 1/2 the resolution of the previous subnetwork. This process loops many times, eventually forming four stages. Since the fourth stage of HRNet was proved to have limited accuracy improvement on the entire model and significantly increased the model parameters [41]. Therefore, to simplify the model structure and reduce the model parameters, we only used HRNet-stage3 for image feature extraction.

#### 2.3.2. Attention Layer

Compared with a Convolutional Neural Network (CNN), the Transformer [42] based on the attention mechanism can obtain stronger global modeling ability, especially when Swin Transformer [43] was proposed recently. This shifted window-based approach can significantly reduce model computation and learn richer representations. In this study, to better capture the constraint relationship between joints, the attention layer was connected behind HRNet-stage3. The attention layer is mainly composed of Patch Embed and Swin Transformer block. Patch embed mainly consists of a norm layer and a convolution (the convolution kernel is 4, the stride is 4, and the output channel is 96), which is used to convert 2D images into 1D sequences for input into the transformer block. For this operation, we refer to the processing method of the Vision Transformer [44]. Firstly, the highest resolution representation from HRNet-stage3 was divided into 24 × 18 patches, with each patch being the size of 4 × 4. Then, each patch was reshaped into a 1D vector and mapped to 96 dimensions. Finally, normalize each patch. After acquiring the 1D sequence, the Swin Transformer block is connected to the Patch Embed to learn the joint point feature representation. Each Swin Transformer is used in pairs, the first block employing the conventional window partitioning strategy, and the second block using the shifted window partitioning strategy. The detailed structure of the Swin Transformer block is shown in Figure 3. It mainly consists of window-based multi-head attention, shift window-based multi-head attention, multi-perceptron, and layer norm, where LN is layer norm, W-MSA and SW-MSA are multi-head self-attention using regular and shifted windows, respectively, MLP is multilayer perceptron, ${\hat{x}}^{l}$ and $x^{l}$ denote the output features of the W-MSA module and the MLP module in the lth block. In this operation, Swin Transformer block is superimposed 4 times, each Swin Transformer block window size is 6, num heads is 48. Please refer to [43] for detailed information on the Swin Transformer.

#### 2.3.3. Heatmap Regression

To obtain the final 2D heatmaps predictions, a transposed convolution (the kernel is 4, the stride is 4, the output channel is 10) was used on the output vector of the Swin Transformer block. The size of the heatmap is 1/4 of the input image. The mean squared error (MSE) is used to calculate the loss between the predicted feature map and the ground truth.

### 2.4. Joint Point Detection of Pig

In this part, we used the improved joint point detection model HRST to localize the joint points of pigs and compared the currently popular joint point detection models such as Simple Baseline [19], HRNet, HRNetv2 [22], and Tokenpose [23] to verify the detection performance of HRST. All joint point detection models were trained, validated, and tested on the same dataset. Furthermore, we combined HRST and CenterNet with DLA-34 as the feature extraction network to detect the joint points of multiple pigs, making it applicable to large-scale farming. Multi-pig joint points detection was carried out through two steps. Firstly, CenterNet with DLA-34 as feature extraction network was used to detect pig posture (lying or standing). Then, HRST was used to detect pig joint points in a standing state.

## 3. Result and Discussion

### 3.1. Posture Detection

#### 3.1.1. Implementation Details

The experimental environment was based on the CentOS7.3 operating system. The hardware was an Intel(R) Xeon(R) Gold 5120 CPU with a Tesla V100-PCIE-32GB graphics card. The programming language was Python 3.8; the deep-learning framework was Pytorch1.8.1. All images were uniformly scaled to 1280 × 720 to reduce memory consumption. In the one-stage object detection algorithm, we used mosaic data augmentation, multi-scale training, and the SGD optimizer with a momentum of 0.937 and weight decay of 5 × 10^−4^. The initial learning rate was set as 1 × 10^−2^. In the two-stage object detection algorithm, we used random horizontal flipping and the SGD optimizer with a momentum of 0.9 and weight decay of 1 × 10^−4^. The initial learning rate was set to 1 × 10^−2^ and then decreased to 1 × 10^−3^ and 1 × 10^−4^ at 70 and 120 epochs. All models terminated training at 210 epochs.

#### 3.1.2. Posture Detection Results

**Figure 4:**
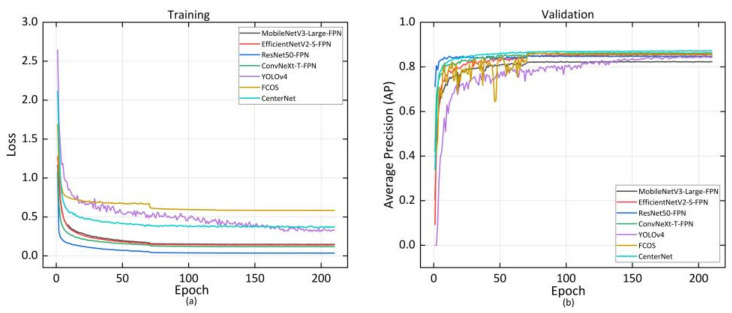
The training loss curve (**a**) and AP curve (**b**) of the posture detection model.

Five common evaluation indicators such as AP, average recall (AR), Giga floating-point operations per second (GFLOPs), model parameters, and frames per second (FPS) were used for comprehensive evaluation of the model performance. AP and AR are calculated from intersection over union (IOU) [45] to evaluate model detection performance. GFLOPs and model parameters are indicators for evaluating algorithm complexity, and FPS is an indicator for evaluating model inference speed. During model training, we recorded the training loss of the model and the AP in the validation set. As we can see from Figure 4a, with the continuous iteration, the loss of the model decreased and converged to a lower value, indicating that the model has been trained. Faster-RCNN with ResNet50-FPN as the feature extraction network had the fastest convergence speed and the lowest loss. Figure 4b shows that the AP of the model in the validation set gradually rose and eventually converged as the number of iterations increased. CenterNet with DLA-34as the feature extraction network converged at the highest accuracy.

To further verify the effect of model training, the trained model was tested in the same test set. Table 3 shows the comparison results of various performance indicators.

**Figure 5:**
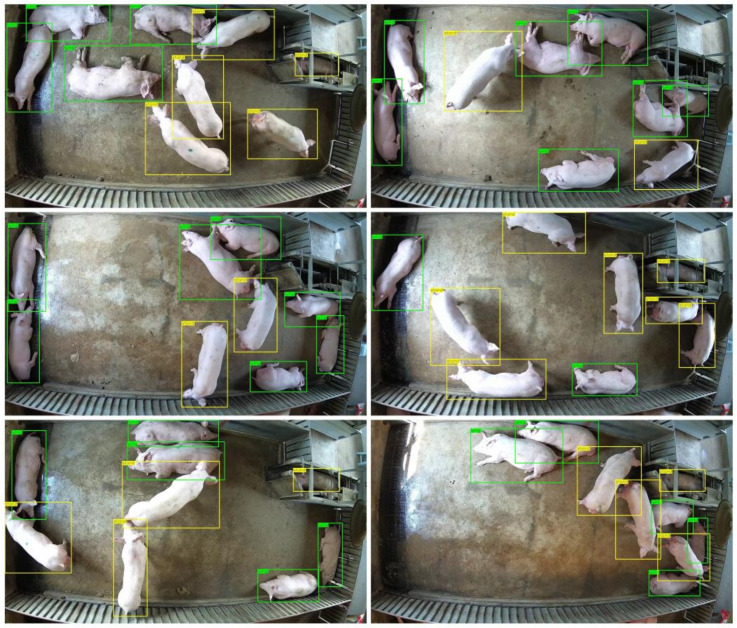
Examples of standing (yellow rectangles) and lying (green rectangles) detected by CenterNet with DLA-34 as the feature extraction network.

The results demonstrate that in the one-stage detection model, the anchor-free CenterNet can accurately detect the posture of the pig and obtain the best detection accuracy (AP of 86.5%). In addition, the anchor-based yolov4 achieved the fastest detection speed at 52 images per second. In the two-stage detection model, ConvNeXt-T-FPN can better extract features and achieved the best detection accuracy (AP of 86.1%). Figure 5 shows the detection results of CenterNet with DLA-34 as the feature extraction network on the posture test set of pigs. As can be seen from the figure, each pig can be identified and wrapped with a bounding box, indicating that our model could accurately distinguish the postures of pigs.

### 3.2. Joint Points Detection

#### 3.2.1. Implementation Details

In joint point detection experiments, all images of the input model were adjusted to 384 × 288 by preprocessing. Additionally, the Adam optimizer was used for random rotation ([−45°, 45°]), random scaling ([0.65, 1.35]), and random horizontal flipping. The initial learning rate was set to 1 × 10^−3^, and then dropped to 1 × 10^−4^ and 1 × 10^−5^ at 600th and 800th epochs, respectively. All models were trained for 1000 epochs.

#### 3.2.2. Joint Points Experimental Results

**Figure 6:**
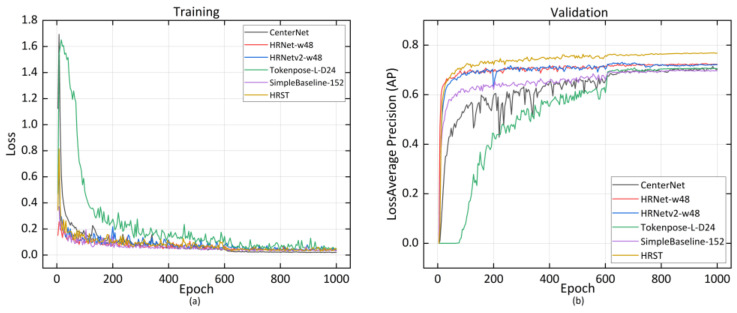
The training loss curve (**a**) and AP curve (**b**) of joint points detection model.

Similar to pig posture detection, AP and AR, GFLOPs, model parameters, and FPS were used for evaluation of the effectiveness of the joint points detection model. AP and AR were computed from Object Keypoint Similarity (OKS) defined in COCO [46]. To describe the training process of the model, we recorded model loss and the mean average precision in the validation set. Figure 6a indicates that the loss gradually decreased and eventually converged to a lower value as the number of iterations increased. Figure 6b shows that the AP in the validation set continued to rise and eventually converged as the number of iterations increased. Finally, our improved joint point detection model HRST converged with the highest AP.

**Figure 7:**
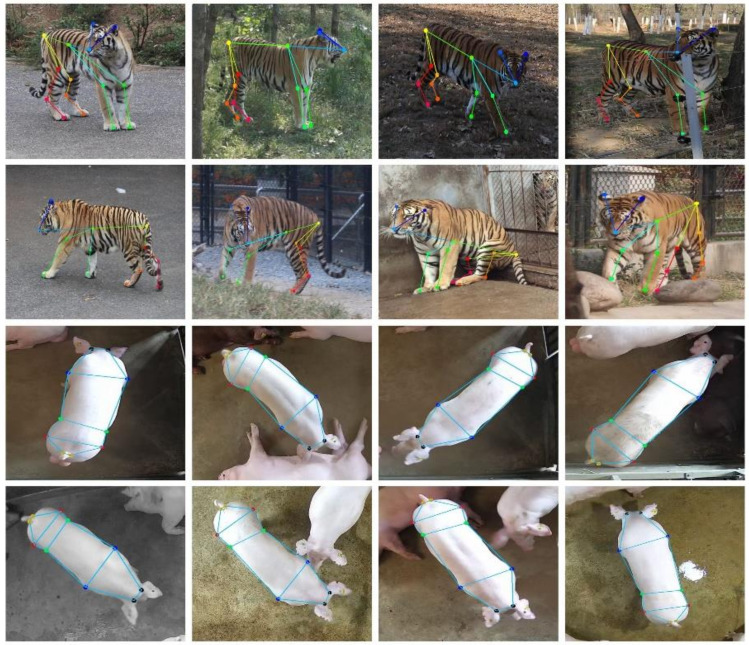
Detection results of HRST on pig and ATRW test sets.

**Figure 8:**
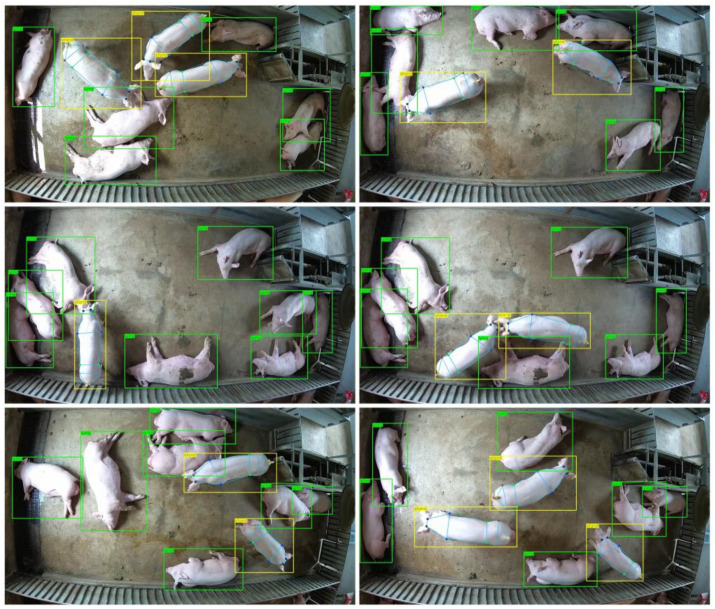
Example of multi-pig joint point detection. The green bounding box represents lying, and the yellow bounding box shows standing. Joint point detection is only performed on standing pigs.

After all joint point model training was completed, the joint point test set of pigs was used to verify the training effect of the model. The comparison of model performance metrics (Table 4) suggested that HRST, our improved joint points detection model, achieved the highest detection accuracy with an AP of 77.4% while having the fewest model parameters of 17.3 M. Compared with HRNet, the GFLOPs and model parameters of HRST reduced by 41.7% and 72.8%, respectively, but the AP increased by 6.8%, indicating that the Swin Transformer block could replace the fourth stage of HRNet to learn more efficient information. Similarly, compared with CenterNet, HRST still has great advantages. It can reduce model parameters by 16% while increasing AP by 10.2%. In addition, HRST also has great advantages in detection speed which reached 40 frames per second. To further validate the generalization performance of HRST, we evaluate it on the public ATRW dataset. The results are shown in Table 5. As the dataset increases, the gap between the AP of the models narrows. However, our improved HRST still achieves the best detection performance (AP of 89.6%) with the fewest model parameters (17.3 M). Figure 7 shows the prediction results of HRST on the test set, where the joint points of each target can be accurately identified. Finally, to better apply to large-scale farms, we combined the trained HRST and CenterNet with DLA-34 as the feature extraction network to detect the joint points of multiple pigs. Figure 8 shows an example of multi-pig joint point detection prediction. As seen from the figure, the posture of each pig can be accurately identified, and the joint points of the standing pig can be detected.

## 4. Conclusions

The accurate positioning of joint points is crucial for body size estimation. In this paper, we employ a top-down approach to detect the joint points of multiple pigs. The pig’s posture was first detected using the object detection method, and then the joint point detection was performed on the standing pig. In pig posture detection, to better detect the posture of pigs, we compared one-stage and two-stage detectors. The results show that CenterNet withDLA-34 as the feature extraction network can accurately identify different pig postures and achieve the highest detection accuracy (AP of 86.5%). In pig joint point detection, since the current joint point detection models focus on improving model accuracy and ignore model parameters and detection speed, we proposed an improved joint point detection model HRST. The model can improve model accuracy while significantly reducing model parameters by replacing the fourth stage of parameter redundancy in HRNet with a Swin Transformer block. The experiments indicated that HRST achieved an AP of 77.4%, which is better than other joint point detection methods. In addition, HRST still has significant advantages in GFLOPs, model parameters, and detection speed compared with the mainstream joint point detection models. Our research provides technical support for accurate and rapid positioning of joint points, which can be applied to large-scale farms to achieve contact-free, stress-free body size estimation of multiple pigs. At the same time, the research can be further improved. First, multi-pig joint point detection is achieved by training two models separately, which is usually inefficient. In addition, we did not investigate whether this joint detection method also performs well in complex scenes (distortion and occlusion). Therefore, further research will be conducted in the following aspects: (1) Based on CenterNet, we will integrate posture and joint point detection into a model to achieve end-to-end training. (2) We will consider augmenting data of pig joints in complex scenes to improve the robustness of the model.

## Figures and Tables

**Table 1:** Details of posture annotation dataset.

Posture Classes	Number of Individual Postures			
	Train Dataset	Validation Dataset	Test Dataset	Total
Standing	5499	735	644	6234
Lying	9058	1084	1171	10,412
Total sample	14557	1819	1815	18,191

**Table 2:** Standard deviation of each joint point.

Joint Point	σ	Joint Point	σ
left neck	0.005322321731521584	right abdomen	0.005311422910349784
right neck	0.00546914966592658	left hip	0.010024322728349425
left shoulder	0.009892777323066001	right hip	0.008588638693752731
right shoulder	0.00871434068851134	left tail	0.004319627728346724
left abdomen	0.004523805890671292	right tail	0.00422832022133345

**Table 3:** Results of different object detection models on the pig posture test set. GFLOPs represent the computational effort of the model; Params represent the number of parameters; AP^50^ and AP^75^ are the average precision when the intersection over union (IOU) threshold is set to 0.5 and 0.75. AP and AR are average precision and average recall averaged over 10 IOU threshold (0.50:0.05:0.95); FPS represents the inference speed. The best results are in bold.

Class	Feature Extractor	GFLOPs	Params	AP	AP^50^	AP^75^	AR	FPS
Faster-RCNN	ResNet50-FPN	177.11	41.4M	84.7	99.0	98.6	88.9	18
Faster-RCNN	MobileNetV3-Large-FPN	**6.62**	**6.2M**	82.3	**99.5**	97.8	86.6	29
Faster-RCNN	EfficientNetV2-S-FPN	59.73	24.3M	84.8	99.0	98.9	88.9	21
Faster-RCNN	ConvNeXt-T-FPN	97.98	34.3M	86.1	**99.5**	**99.0**	90.0	18
YOLOv4	CSPDarknet53	119.50	63.9M	84.1	98.5	97.8	88.1	**52**
FCOS	ResNet50-FPN	177.47	31.84M	85.7	99.0	98.1	**90.2**	21
CenterNet (for posture detection)	DLA-34	96.29	20.2M	**86.5**	99.0	98.9	89.5	26

**Table 4:** Results of different joint point detection models on the joint point test set of pigs. AP^50^ and AP^75^ are the average precision when Object Keypoint Similarity (OKS) threshold is set to 0.5 and 0.75. AP and AR are average precision and average recall averaged over 10 OKS threshold (0.50:0.05:0.95). We calculated the percent difference in params, GFLOPs, and AP between models marked with the same symbol. "†" marks the models we compared, and "↓" and "↑" indicate the decline and increase of the comparison results, respectively.

Class	GFLOPs	Params	AP	AP^50^	AP^75^	AR	FPS
CenterNet (for joint point detection)	**13.95**	20.6M	67.2	94.4	83.2	73.8	**56**
HRNet-w48	35.43 †	63.6M †	70.6 †	94.9	86.4	78.0	26
HRNetv2-w48	39.53	65.9M	72.1	**96.6**	**91.7**	78.6	24
Simple Baseline-152	28.67	68.6M	69.6	96.0	86.6	76.0	48
Tokenpose-L-D24	23.98	29.9M	70.2	95.6	85.1	77.6	26
HRST	20.65 † (↓41.7%)	**17.3 M** † **(** ↓**72.8%)**	**77.4** † **(** ↑**6.8%)**	95.9	90.4	**82.8**	40

**Table 5:** Results of different joint point detection models on the ATRW test set. The relevant indicators of this table are consistent with those in Table 4.

Class	GFLOPs	Params	AP	AP^50^	AP^75^	AR	FPS
CenterNet (for joint point detection)	**13.95**	20.6M	75.2	95.2	77.0	86.5	**56**
HRNet-w48	35.43 †	63.6M †	88.8 †	97.2	90.6	91.9	26
HRNetv2-w48	39.53	65.9M	89.0	97.3	90.4	92.2	24
Simple Baseline-152	28.67	68.6M	86.4	96.4	90.3	90.1	48
Tokenpose-L-D24	23.98	29.9M	87.1	97.2	89.3	90.6	26
HRST	20.65 † (↓41.7%)	**17.3M** † **(** ↓**72.8%)**	**89.6** † **(** ↑**0.8%)**	**98.4**	**91.5**	**92.4**	40

## Data Availability

The code is publicly available at https://github.com/XiaopinWang/HRST.git (accessed on 25 July 2022). The image data are publicly available at https://drive.google.com/drive/folders/1nlNuPAn8pSCkUudYHnOpYpd8rB9NtLoY?usp=sharing (accessed on 25 July 2022).
